# Evaluation of Wild Flora Surrounding Blueberry Fields as Viable Hosts of *Drosophila suzukii* (Matsumura, 1931) (Diptera: Drosophilidae) in Georgia

**DOI:** 10.3390/insects12080667

**Published:** 2021-07-22

**Authors:** Joshua A. Grant, Ashfaq A. Sial

**Affiliations:** Department of Entomology, University of Georgia 413 Biological Sciences Building, Athens, GA 30602, USA; joshua.grant25@uga.edu

**Keywords:** spotted-wing drosophila, host reservoirs, wild host, alternative host, susceptibility, viability

## Abstract

**Simple Summary:**

Understanding an agricultural pest’s biology and ecology is essential for the creation of integrated pest management strategies. To elucidate what wild fruit resources the invasive blueberry pest, spotted-wing drosophila, can use to successfully reproduce, we conducted choice and no-choice laboratory assays. In our experiments, the fly was able to lay eggs in and develop to adulthood in eight of the eighteen fruiting plant species common in woodlands adjacent to commercial blueberry fields. Though none of these eight viable hosts were found to be preferred when the fly was given the choice between it and a commercial blueberry, the identified hosts could still be used by the drosophilid to maintain populations during as well as outside the blueberry growing season. Listing viable hosts better informs future studies and growers on how to balance pest management strategies targeting these viable fruit hosts while maintaining these marginal landscapes.

**Abstract:**

*Drosophila suzukii*, an economically important pest of small and thin-skinned fruits, has caused annual crop losses up to 20% in the state of Georgia’s multimillion-dollar blueberry industry. The known host range of *D. suzukii* is large, yet the breadth of uncultivated and wild plants that can serve as alternative hosts in the southeastern United States is still not fully understood. Establishing comprehensive lists of non-crop *D. suzukii* hosts in woodlands near blueberry production will assist in the creation of more sustainable integrated pest management (IPM) strategies. Objectives of this study were to determine viability of wild fruiting plant species to this pest based on survivorship to adulthood and assess *D. suzukii* short-range preference between cultivated blueberries and wild fruit. Laboratory choice and no-choice assays were performed to determine if *D. suzukii* could complete its development on wild fruits sampled from the field. Results from our no-choice assays indicated that multiple species of wild fruits surveyed in Georgia were viable *D. suzukii* hosts including blackberry species, deerberry, hillside blueberry, common pokeweed, beautyberry, elderberry, evergreen blueberry, and large gallberry. Yet, none of these hosts were preferred by adult female *D. suzukii* as ovipositional substrates when compared to cultivated blueberries. However, these uncultivated species have the potential to sustain *D. suzukii* populations pre- and post-harvest season. This information can help farmers do more targeted management of these viable alternative hosts from wooded areas surrounding blueberry fields in order to minimize *D. suzukii* populations.

## 1. Introduction

The invasive Asian vinegar fly, *Drosophila suzukii* (Matsumura, 1931) (Diptera: Drosophilidae), commonly referred to as spotted-wing drosophila (SWD), is now a global pest of soft-skinned fruits as it has recently expanded its range across Europe and the Americas [1,2,3,4,5]. The first continental United States trap captures of this pest occurred in California in 2008 [3,6] and it is now established in all economically important regions for fruit production in North America [7]. Like other *Drosophila*, *D. suzukii* use fruits to complete its life cycle and also as adult food resources [8,9]. Unlike other *Drosophila* Fallén, 1,823 species of this fly can oviposit into fresh pre-harvest fruit [2], causing damage before fruits are even marketable. Such damage is caused by oviposition wounds, internal feeding of maturing *D. suzukii* larvae, and secondary infections [2,3,10,11,12,13]. Adult female *D. suzukii* have a heavily sclerotized ovipositor which allows them to lay eggs in ripening and ripe fruit [2,13,14,15]. Economic losses are furthered by the current management costs of weekly broad-spectrum insecticide sprays and the zero-tolerance policy in the market place leading to rejection of entire shipments of fruit due to the presence of *D. suzukii*-infested fruit [1,16,17,18].

One group that is threatened by *D. suzukii*-related damage is blueberry (*Vaccinium* spp. L.) growers in the southeastern United States. Blueberry production has surged over the past 15 years in this region; the state of Georgia being a prime example where approximately 30,166 acres of blueberries were harvested in 2018, worth over US$300.3 million [19]. Other valuable cultivated fruit crops including plums, figs, cherries, blackberries, strawberries, and raspberries have also been reported to be susceptible to *D. suzukii* infestation to some degree [1,2,4,20,21,22]. Such susceptible cultivated fruit can be planted in the vicinity of one another in mixed fruit orchards [22], such as muscadine grapes in South Georgia blueberry farms and can harbor *D. suzukii* for longer durations [23,24,25]. The fruit species of interest in this study, however, were the common wild berries found ripening in the bordering woodlands adjacent to blueberry fields in Georgia.

Past trapping data from southeast Georgia has revealed that, on average, *D. suzukii* are captured more readily in the woodlands surrounding blueberry fields than among the berry crop itself [26]. A similar trend has been seen in other cropping systems such as cherry orchards [27] and vineyards [28]. The habitat parameter this study focuses on as a possible driver for this trend is availability of susceptible wild fruit. It is known that a multitude of uncultivated/non-crop, wild, native, invasive, and/or ornamental plants can act as hosts for *D. suzukii*, collectively called alternative hosts. The list of identified viable plant hosts has been growing as alternative host studies are conducted wherever this pest expands its range [2,20,29,30,31,32,33,34,35,36]. The challenge remains that, with these comprehensive lists of *D. suzukii* wild hosts, some may also be beneficial as habitat for natural enemies and pollinators [9,37]. A balance must be struck between targeting viable hosts of *D. suzukii* and maintaining natural habitat for other organisms providing ecosystem services.

Alternative hosts can facilitate and/or maintain higher populations of *D. suzukii* within an agricultural landscape in multiple ways, which is why first assessing which plants can serve as reservoirs will allow more efficient integrated pest management efforts around such source material [9,34]. Recent trapping and mark-recapture studies have shown that *D. suzukii* can move over 100 m, indicating that movement between field margins and berry crops is possible for this pest [34,38,39]. This suggests that woodlands with ripe susceptible fruit could buffer against management practices such as pesticide sprays that only target the production fields. Alternative hosts could maintain *D. suzukii* presence near agricultural landscapes during and after the cultivated crop growing season [9,27,37,40].

Based on the economic damage caused by *D. suzukii* and the potential role of numerous wild plant species as *D. suzukii* hosts, an assessment of common fruiting plants surrounding blueberry fields in Georgia is necessary. Thus, the objectives of this study were to determine viability of wild fruiting plant species occurring in woodland habitats surrounding blueberry fields as hosts of *D. suzukii* based on oviposition and survivorship to adulthood, and assess *D. suzukii* short-range preference between cultivated blueberries and wild fruit.

## 2. Materials and Methods

### 2.1. Sampling

Fruit sampling was performed at nine sites across southeast Georgia counties known for high blueberry production (Table 1). Samples were collected from fruiting plant species in woodlands adjacent to blueberry fields and sites were visited weekly during the summers (May–August) of 2015 and 2016. In between these growing seasons, samples were collected on a bi-weekly basis. Depending on berry size, samples consisted of approximately 70–100 fruits picked from plants, collected in 16 oz plastic deli containers (Choice Plastics, Mound, MN) marked with location information and brought back to the lab for assays. Positive identifications of wild plant species were aided by reference guides. The following 18 plant species were collected and assessed in the lab for this study: American beautyberry, *Callicarpa americana* L.; blackberry spp., *Rubus* spp. L.; cat sawbrier, *Smilax glauca* Walt.; Chinese privet, *Ligustrum sinense* Lour.; common pokeweed, *Phytolacca americana* L.; deerberry, *V. stamineum* L.; elderberry, *Sambucus canadensis* L.; evergreen blueberry, *V. myrsinites* Lam.; hillside blueberry, *V. pallidum* Aiton.; lanceleaf greenbrier, *Smilax smallii* Morong.; large gallberry, *I. coriacea* (Purch) Chapm.; muscadine grape, *Vitis rotundifolia* Michx.; myrtleleaf holly, *I. myrtifolia* Walt.; red bay, *Persea borbonia* (L.) Spreng.; red chokeberry, *Aronia arbutifolia* (L.) Pers.; saw palmetto, *Serenoa repens* (W. Bartram) Small; gallberry, *I. glabra* (L.) A. Gray; and Virginia creeper, *Parthenocissus quinquefolia* (L.) Planch.

### 2.2. Insect Rearing

Adult *D. suzukii* flies of both sexes used in this study were 5–7 days old and were reared as part of a laboratory colony in Clarke County, GA. The colony was established using specimens captured from wild populations in the same county during the summer of 2013. Cultures were maintained in 177-mL plastic square bottomed bottles (Genesee Scientific, San Diego, CA, USA) where flies were provided ~50 mL of standard diet (65.1 g cornmeal, 13.0 g yeast, 6.8 g agar, 55.0 mL molasses, 14.5 mL tegosept, and 2.4 g propionic acid salt, per 1.1 L dH_2_O), as discussed in Jaramillo et al. (2015). The lab colony of *D. suzukii* were kept in incubators (Model I36VLCB, Percival Scientific, Perry, IA, USA) at 24 ± 1 °C, 65 ± 5% relative humidity, and a photoperiod of 14:10 h (L:D).

### 2.3. Laboratory Assays

To assess the susceptibility of wild fruit to *D. suzukii* infestation, ripe fruit collected from woodlands bordering blueberry production were introduced to laboratory reared flies in choice and no-choice assay chambers. All collected fruit used in choice and no-choice assays were washed with dH_2_O and found under a dissecting microscope to be free of *D. suzukii* oviposition holes and wounds prior to use. Chambers consisted of 8oz plastic deli cups (Choice Plastics, Mound, MN, USA) with a 1/2 inch sand substrate, vented cover, and a wetted cotton ball which provided moisture to the flies. Berries rested on the sand substrate, which minimized mold growth and prevented the berries from rolling within the chambers. Two 5–7-day-old male and two 5–7-day-old female *D. suzukii* adults were introduced into the chambers for 48 h during the summer of 2015 and for 24 h in 2016. Assays were set up in laboratory settings of 24 ± 1 °C with a 70% relative humidity, where lighting was diffuse and ran on an approximately 14:10 h (L:D) cycle. Ten replications per trial for each species and assay type were run per sample collection and control assays using ripe cultivated blueberries were run concurrently with other no-choice assays. The number of wild plant berries used per cup in both assay types varied depending on berry size to match the approximate volume of cultivated blueberries. All collected fruit was used in laboratory trials within two days of collection date.

No-choice assays were run with one species of fruit per cup. Choice assays were run with one cultivated blueberry and a number of berries of one wild fruit species, as mentioned above. Both species were placed equidistant from the cotton ball in choice assay chambers. After each exposure time (48 or 24 h), flies were aspirated out of assay chambers and eggs oviposited were counted under a dissecting microscope. The number of eggs were recorded by observing the egg breathing filaments which protrude out from each oviposition hole on the surface of the infested fruit. Fruit were then transferred to vented 2 oz plastic portion cups (Fabri-kal, Kalamazoo, MI, USA) lined with two layers of paper towel to absorb any berry leakage. Berries were then left to rear for three weeks under the same laboratory settings as mentioned above, after which any eclosion to adulthood was also recorded. The remaining fruit not used in our choice and no-choice assays were reared in vented 8oz plastic cups to assess natural infestation levels per tested fruiting species by recording any fly eclosion to adulthood after three weeks.

### 2.4. Data Analysis

The assay chambers were the experimental unit in this study and the response variable was the *D. suzukii* count/berry/time exposure. In this study, susceptibility meant that oviposition occurred in the fruit and that at least one larva completed development to adulthood. No-choice oviposition and adult eclosion data (count/berry/time exposure) were log10(x + 1) transformed to meet the assumptions of normality and run through one-way ANOVA (Fit X by Y Routine, JMP Pro 13). No-choice egg and adult count data were analyzed by year and pooled for each plant species tested. Choice oviposition data (eggs/berry) were log10(x + 1) transformed to meet the assumptions of normality and analyzed using one-way ANOVA (Fit X by Y Routine, JMP Pro 13) by year and for each plant species confirmed to be susceptible in no-choice assays. The proportion of eggs deposited in alternative hosts versus cultivated ripe blueberries (eggs in alternative berries [eggs in alternative berries + eggs in cultivated blueberries]) were analyzed using one-way ANOVA and Student’s *t*-test (JMP Pro 13. Survivorship data were analyzed using one-way ANOVA (Fit X by Y Routine, JMP Pro 13) and reported as proportion of eggs that successfully completed the development to adult eclosion in no-choice assays. Significance was determined for the fixed effects at α = 0.05. All means were separated using Tukey–Kramer honestly significant difference tests and were back transformed for data presentation [41].

## 3. Results

### 3.1. No-Choice Assays

**Figure 1:**
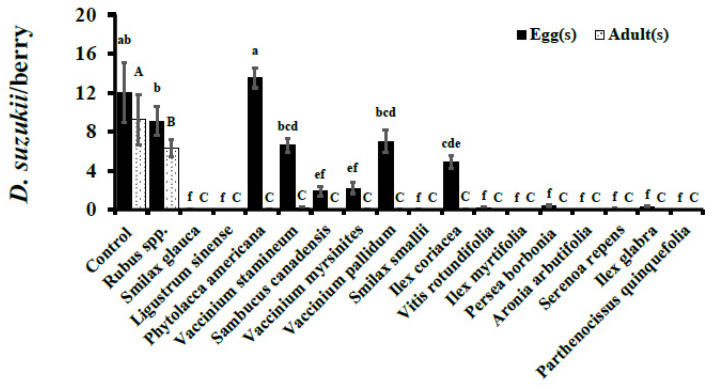
**No-choice Assays.***D. suzukii* oviposition after 48 hr exposure and adult eclosion per berry (Mean ± SEM) in 2015. Oviposition (F_17, 900_ = 59.59, *p* < 0.0001) and adult eclosion (F_17, 900_ = 73.94, *p* < 0.0001) analyzed using one-way ANOVA. Within a given developmental stage (egg or adult) bars with the same letter are not significantly different (*p* ≥ 0.05).

**Figure 2:**
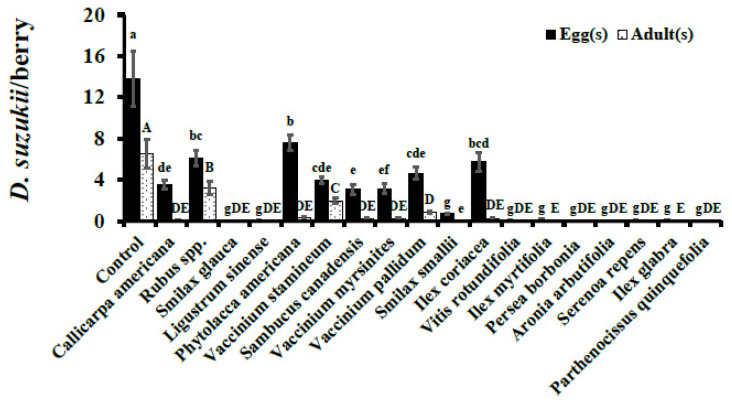
**No-choice Assays.***D. suzukii* oviposition after 24 hr exposure and adult eclosion per berry (Mean ± SEM) in 2016. Oviposition (F_18, 810_ = 102.08, *p* < 0.0001) and adult eclosion (F_18, 810_ = 53.04, *p* < 0.0001) analyzed using one-way ANOVA. Within a given developmental stage (egg or adult) bars with the same letter are not significantly different (*p* ≥ 0.05).

Oviposition (number of eggs/berry) observed in cultivated blueberry controls was significantly higher than in wild fruits assayed in 2015 except *P. americana*, *Rubus* spp., *V. stamineum*, and *V. pallidum* (F_17, 900_ = 59.59, *p* < 0.0001; Figure 1). Similarly, in 2016, cultivated blueberry controls were the preferred ovipositional substrate having statistically higher oviposition than wild fruits (F_18, 810_ = 102.08, *p* < 0.0001; Figure 2). In 2015, no oviposition was observed in *I. myrtifolia*, *Ligustrum* spp., *A. arbutifolia*, and *P. quinquefolia*. Similarly, no eggs were deposited in *S. glauca*, *P. borbonia*, *A. arbutifolia*, and *P. quinquefolia* during 2016. Oviposition in wild fruits in 2015 ranged from 6.59 ± 0.69 (*V. stamineum*) to 13.53 ± 1.03 (*P. americana*), though the overall *D. suzukii* adult emergence was low in comparison (Figure 1). For example, *P. americana* berries were infested most heavily by *D. suzukii* larvae of all the wild berries assayed in 2015 but had adult eclosion of only 0.04 ± 0.02 adults per berry (Figure 1). Similar trends of high oviposition but minimal-to-no adult eclosion were also observed in other wild fruits in 2016 (Figure 2). 

Cultivated blueberry controls had statistically higher adult eclosion than any of the tested wild berries in both years (2015: 9.20 ± 2.59, F_17, 900_ = 73.94, *p* < 0.0001; 2016: 6.50 ± 1.38, F_18, 810_ = 53.04, *p* < 0.0001) (Figure 1 and Figure 2). Of all the wild berries assayed, only *Rubus* spp. had adult eclosion of more than one fly per berry (6.33 ± 0.87) in 2015, whereas in 2016 both *Rubus* spp. (3.22 ± 0.61) and *V. stamineum* (1.98 ± 0.23) had adult eclosion above one fly per berry (Figure 1 and Figure 2). Other wild berries in which survivorship was observed in 2015 included *V. stamineum*, *P. americana*, *V. myrsinites*, and *I. coriacea*, with 0.04 ± 0.03, 0.02 ± 0.01, 0.01 ± 0.01, and 0.01 ± 0.01, respectively (Table 2).

Survivorship of *D. suzukii* to adulthood was significantly higher in *Rubus spp.* than all of the other wild species assayed, but was statistically similar to the cultivated blueberries in both 2015 (F_13, 362_ = 19.37, *p* < 0.0001) and 2016 (F_14, 361_ = 8.46, *p* < 0.0001) (Table 2). Based on survivorship to adulthood, the wild plant species including *Rubus spp*., *V. stamineum*, *V. pallidum*, *P. americana*, *C. americana*, *S. canadensis*, *V. myrsinites*, and *I. coriacea* were found to be viable hosts of *D. suzukii* in our no-choice tests. The rest of the wild plant species assayed in this study had no development to adulthood, and hence were not considered viable hosts of *D. suzukii*.

### 3.2. Choice Assays

**Figure 3:**
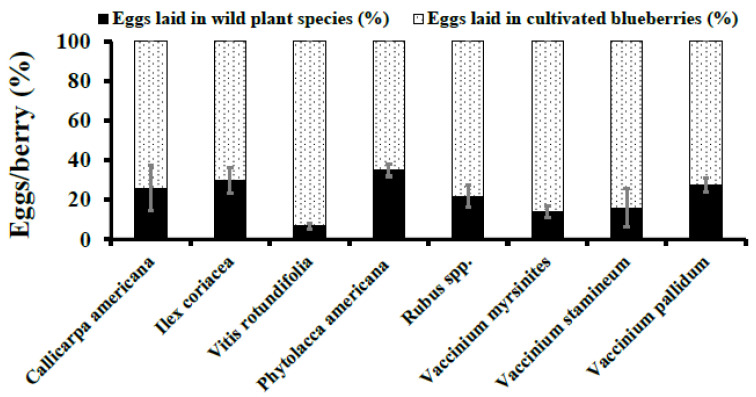
**Choice Assays.***D. suzukii* oviposition per berry (Mean ± SEM) in wild berries were compared to ripe cultivated blueberries in 2015. Student’s *t*-test was used to compare oviposition into wild berries compared to ripe cultivated blueberries (*p* ≥ 0.05).

**Figure 4:**
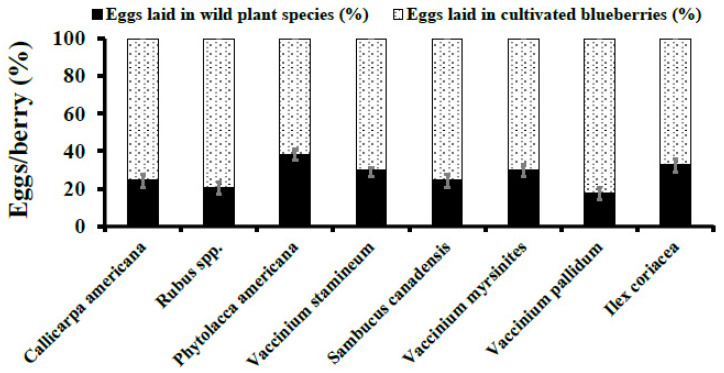
**Choice Assays.***D. suzukii* oviposition per berry (Mean ± SEM) in wild berries were compared to ripe cultivated blueberries in 2016. Student’s *t*-test was used to compare oviposition into wild berries compared to ripe cultivated blueberries.

These tests indicated that adult female *D. suzukii* preferred to oviposit in ripe cultivated blueberries when compared to the wild susceptible fruits assessed in this study. In both 2015 and 2016, significantly more eggs were laid in cultivated blueberries than *C. americana, Rubus spp.*, *P. americana*, *V. stamineum*, *S. canadensis*, *V. myrsinites*, *V. pallidum*, *I. coriacea*, and *V. rotundifolia* (Figure 3 and Figure 4). When comparing oviposition across fruiting species, *P. americana* (4.97 ± 0.59) was statistically similar to all other tested wild plant species except *V. rotundifolia* (0.70 ± 0.24; F_8, 270_ = 3.95, *p* = 0.0002) in 2015.

In 2016, oviposition of *D. suzukii* eggs in *P. americana* berries (8.10 ± 3.06) was statistically similar to *V. stamineum, I. coriacea, and V. myrsinites* and significantly higher when compared to *Rubus spp*., *S. canadensis*, *V. pallidum*, and *C*. *americana* (F_7, 190_ = 3.88, *p* = 0.0006). The proportion of *D. suzukii* eggs deposited into cultivated blueberries was statistically higher than all wild fruit species except *I. coriacea* (F_1, 40_ = 3.89, *p* = 0.0558) (Figure 3 and Figure 4). 

### 3.3. Natural Infestation

Berries taken from the field and reared in the laboratory setting had minimal *D. suzukii* infestation based on adult eclosion through this study. In 2015, a total of 5 *D. suzukii* flies were reared from *S. canadensis* fruit, 15 from *P. Americana*, and 22 flies from *Rubus spp*. These three species, *P. americana*, *S. canadensis,* and *Rubus spp*., also had natural infestation in 2016 with a total of 4, 8, and 35 flies reared from them, respectively. The remaining assessed species, though many were found to be susceptible in laboratory assays, were not infested in the field.

## 4. Discussion

Through our no-choice assays, we identified eight fruiting plant species of the eighteen tested in which *D. suzukii* could complete its life cycle to adulthood: *Rubus* spp., *V. stamineum*, *V. pallidum*, *P. americana*, *C. americana*, *S. canadensis*, *V. myrsinites*, and *I. coriacea*. Of these plants, *Rubus* spp. and *P. americana* had been recently described as susceptible species in multiple other studies [29,42]. Furthermore, previous studies have found other species in the same genera as *I. coriacea*, *V. stamineum*, *V. pallidum,* and *S. canadensis* to be susceptible to *D. suzukii* infestation [9,37,43]. To our knowledge, this study is the first to indicate that *C. americana* can act as a viable host for *D. suzukii*. The plant species mentioned above are listed as viable hosts because at least one instance of oviposition occurred (though usually multiple eggs were deposited) and at least one instance of an adult eclosing out of fruit of each species occurred during this study in 2015–2016. However, there is a disconnect between these no-choice data and the natural infestation levels observed in the field-collected fruit [2,18,20]. Of the susceptible berries picked from the field and reared in the lab, only *Rubus* spp., *S. canadensis*, and *P. americana* had natural infestation, and all infestation levels were low in the single digits. 

This discrepancy between susceptibility in lab yet zero infestation in the field for *V. stamineum*, *V. pallidum*, *C. americana*, *V. myrsinites*, and *I. coriacea* could be due to many factors. The size, density, and dispersal potential of *D. suzukii* populations in the field setting can be drastically affected by fragmentation in landscape vegetation and diversity of host plant choices. Woodlands surrounding blueberry production in the state of Georgia rarely exceed 100 m in any direction but the spacing between these fragmented woodlands is commonly only 10–50 m, which is well within the dispersal distance known for this pest [34,38,39]. Due to this, as well as the large diversity of fruiting herbaceous plants covering the understory of these planted pine habitats providing multiple host choices, the activity of *D. suzukii* could be spread thin throughout this landscape, leading to the small amounts of natural infestation observed in our field-collected wild fruit samples [44].

In the state of Georgia, planted pine habitat is common surrounding blueberry production, which can become overgrown with herbaceous fruiting plants if left unmanaged. These spaces might provide microclimates with favorable conditions for *D. suzukii* during the summer and winter months. Average ambient temperatures experienced during Georgia summers exceed the maximum limits for *D. suzukii* activity, suggesting that food and ovipositional resources need to be within such favorable microclimates [45,46,47,48,49]. Stockton et al. [50] found that not only can fruit be used as a food source for feeding life stages of *D. suzukii*, but so can common forest resources, such as mushrooms and bird manure [50], as well as floral resources for *D. suzukii* adults [51]. Further assessment of these potential *D. suzukii* resources must be conducted to better understand how the marginal landscape surrounding agricultural fields affect the population and distribution of this invasive insect pest. Plotting viable wild host ripening periods against year-round *D. suzukii* activity data could aid in creating infestation models as relevant factors [52].

In addition, possible physical limitations preventing *D. suzukii* oviposition in the field based on characteristics of fruit quality might cause differing results in susceptibility. The skin resistance of grapevine berries was shown to be the determining factor in the number of eggs oviposited when compared to other fruit parameters [53]. Skin penetration resistance and other characteristics such as brix, pH, skin firmness, and coloration were not quantified in this study but have been shown to be major factors in the susceptibility of fruit to pest infestation [29,54,55,56]. On average, it is established that the skin penetration resistance of a fruit is negatively correlated to *D. suzukii* oviposition levels and that fruit with higher pH and brix are more attractive to *D. suzukii* for oviposition and development [20,29,46,55,57,58,59]. It is plausible that natural infestation was underrepresented by the data due to our sampling methods which only focused on fresh fruit off the plant, neglecting the softer and more over-ripe berries fallen onto the ground. Nonetheless, these physical attributes of the berries might be altered once removed from the plant and placed into the laboratory environment.

Oviposition in no-choice assays was consistently high for species such as *P. americana*, *I. coriacea*, and *V. pallidum,* yet minimal development to adulthood was observed. Many challenges in these tests could account for such a trend. The fruit of plants producing smaller berries, such as elderberry, were occasionally seen drying out within the assay chambers before completion of the tests. Furthermore, fungal hyphae growth on *Rubus* spp., even in the dry sand substrate of our choice and no-choice assay chambers, was seen throughout the study, leading to lower survivorship ratios than expected [60]. Additionally, Olazcuaga et al. [61] assessed 12 fruits for ovipositional preference and larval performance showed that these two metrics of performance do not equal each other [61].

In 2016, due to the small ratios of eggs to adult eclosion, the exposure time for all laboratory assays was halved from the previous year to 24 h because of the possibility of intraspecific competition between the larvae. Though oviposition rates were mostly consistent from 2015 to 2016, *D. suzukii* survivorship did increase in plant groups such as *Rubus* spp., *P. americana*, and *V. stamineum*. However, the wild plant species that are attractive as ovipositional substrates and that do not yet support *D. suzukii* adult development will act as egg sinks for this pest and will not contribute to their populations [9]. Plant species which act as sinks could be beneficial in reducing *D. suzukii* numbers if occurring in riparian zones around cultivated fields, functioning like a dead-end trap plants, especially if they reduce the infestation levels in the more attractive cultivated crop as Ulmer et al. [62] found in lab assays with strawberries [62]. Interestingly, other species in the same family as *C. americana*, Lamiacae (mint family), produce essential oils that have insecticidal properties when administered as fumigants and are toxic on contact to *D. suzukii* [63]. All of the challenges mentioned thus far about short-range choice and no-choice chambers indicate that controlled laboratory assays are not ultimate representations of fruit susceptibility and should be paired with field surveying to create more definitive conclusions of host potential.

None of the alternative hosts assessed were preferred by adult female *D. suzukii* compared to ripe cultivated blueberries, when choosing where to oviposit their eggs. These findings align with Rodriguez-Saona et al. [43] results which indicate the cultivated blueberries are preferred as ovipositional substrates over wild blueberry species [43]. This short-range preference suggests that these viable hosts may act as reservoirs to *D. suzukii* populations before and after the blueberry growing season, when the most attractive fruits are in the bordering woodlands and not the agricultural fields. In Georgia, this blueberry fruit availability spans from late April through mid-July with the majority of ripe and/or fallen overripe fruit present from mid-to-late-summer. The inverse situation, where alternative hosts are preferred over cultivated fruit, might increase populations of *D. suzukii* around berry crops but could also lure the populations away from the farm and into the woodlands [9]. These viable hosts might serve as overwintering refuges and/or adult food sources during times of extreme climatic conditions [27,40,64,65,66]. In either case, alternative hosts that are found to be growing in woodlands adjacent to blueberry production in Georgia should be one focus of the IPM programs, and understanding the temporal and geographical interactions of these fruits with *D. suzukii* activity within the growing season is critical [67]. Fitness and ovipositional preference tests run by Diepenbrock et al. [18,42] indicate that the choice of adult female *D. suzukii* to lay eggs in one berry versus another may also be influenced by natal host species [18,42]. By using flies reared from both *Rubus spp.* and *P. americana*, they showed that flies prefer to oviposit their eggs in the same species from which they emerged and that this has implications on their fitness [18,42]. Such a trend should be kept in mind when creating future host use assays.

## 5. Conclusions

This was the first comprehensive study to address alternative hosts of *D. suzukii* in the state of Georgia. Taken together, choice, no-choice, and field infestation numbers, this study has revealed eight species of wild fruiting plants ubiquitous around Georgia blueberry production to be susceptible to *D. suzukii.* This is the first report of *C. americana* being identified as a viable host of *D. suzukii* while the remaining species belong to genera with known hosts. It is not surprising that three of the susceptible species were in the same genus, *Vaccinium*, as cultivated blueberries grown in this region. These data also indicate that these alternative hosts are most likely sustaining *D. suzukii* populations during and after the blueberry season, but are likely not increasing the fly numbers in season due to cultivated blueberries being a preferred host. Future studies should further address the susceptibility of wild fruiting plants around blueberry fields and other agricultural settings by using field and laboratory studies in tandem. Understanding the role of not only the physical properties of the wild berries but also chemical cues from those wild berries in ovipositional site selection by *D. suzukii* will contribute more to the body of work surrounding alternative hosts [67]. Research intended to elucidate the dispersal capabilities of *D. suzukii* could be implemented in the blueberry cropping systems during harvest season and pre- and post-harvest to track the *D. suzukii* movement and correlate it to berry presence, not just trap captures [68]. These data will assist in the formation of protocols for such studies and can aid growers immediately by indicating what plant species to target with IPM strategies. Such control measures can be site-specific and can be applied to the woodland landscape in spot treatments, targeting only *D. suzukii* viable hosts and reducing the loss of beneficial plants by creating refuge for natural enemies and pollinators. Plants found to be susceptible in this study can be minimized around blueberry production through physical removal, controlled burns and/or herbicides; however, further research is needed to assess effectiveness of these methods in controlling *D. suzukii* populations and their impact on forest and pollinator health.

## Figures and Tables

**Table 1:** GPS Coordinates for berry collection sites located in six counties in southeastern Georgia, U.S.A.

County	Latitude (N)	Longitude (W)
Appling	31.75317	−82.44336
Bacon	31.51020	−82.45230
Brantley	31.18022	−82.00486
Clinch	30.94030	−82.68980
Pierce	31.45169	−82.17831
Ware	31.15472	−82.59180

**Table 2:** *D. suzukii* Survivorship: Proportion of eggs developing to adulthood (Mean ± SEM) in no-choice assays.

Species	2015		2016	
	*n*	Survivorship	*n*	Survivorship
*Callicarpa americana* L.			28	0.02 ± 0.01 b
*Rubus* spp. L.	54	0.69 ± 0.14 ab	46	0.72 ± 0.14 a
*Smilax glauca* Walt.	2	0.0 ± 0.0 c		
*Ligustrum sinense* Lour.			1	0.0 ± 0.0 b
*Phytolacca americana* L.	97	0.02 ± 0.01 c	49	0.05 ± 0.02 b
Control	8	0.82 ± 0.16 a	10	0.57 ± 0.09 ab
*Vaccinium stamineum* L.	69	0.04 ± 0.03 c	50	0.66 ± 0.08 a
*Sambucus Canadensis* L.	20	0.0 ± 0.0 c	43	0.15 ± 0.06 b
*Vaccinium myrsinites* Lam.	21	0.01 ± 0.01 c	27	0.11 ± 0.05 b
*Ilex glabra* (L.) A. Gray	6	0.0 ± 0.0 c	4	0.0 ± 0.0 b
*Vaccinium pallidum*	27	0.0 ± 0.0 c	47	0.32 ± 0.06 b
*Smilax smallii* Morong.	1	0.0 ± 0.0 c	2	0.0 ± 0.0 b
*Ilex coriacea* (Purch) Chapm.	46	0.01 ± 0.01 c	46	0.09 ± 0.04 b
*Vitis rotundifolia* Michx.	4	0.0 ± 0.0 c	1	0.0 ± 0.0 b
*Ilex myrtifolia* Walt.			5	0.0 ± 0.0 b
*Persea borbonia* (L.) Spreng.	6	0.0 ± 0.0 c		
*Serenoa repens* (W. Bartram) Small	1	0.0 ± 0.0 c	2	0.0 ± 0.0 b

Within a given year, means followed by the same letter are not significantly different (*p* ≥ 0.05).
